# Educational Resilience During the COVID-19 Pandemic: A Descriptive Evaluation of Medical School and Licensing Examination Policies in Japan

**DOI:** 10.7759/cureus.98661

**Published:** 2025-12-07

**Authors:** Takahiro Maruyama, Yudai Kaneda, Sota Sugiura, Tetsuya Tanimoto, Akihiko Ozaki

**Affiliations:** 1 School of Medicine, International University of Health and Welfare, Tokyo, JPN; 2 Medical Education, Clinical Training Center, Jyoban Hospital, Tokiwa Foundation, Iwaki, JPN; 3 School of Medicine, University of Tokyo, Tokyo, JPN; 4 Internal Medicine, Navitas Clinic, Tokyo, JPN; 5 Surgery/Breast Surgery, Jyoban Hospital, Tokiwa Foundation, Iwaki, JPN

**Keywords:** covid-19, education equity, examination, japan, medical students

## Abstract

Introduction: The COVID-19 pandemic significantly disrupted medical education worldwide, highlighting critical vulnerabilities in educational continuity, especially in the context of high-stakes examinations. This study investigates how medical schools and national examination authorities in Japan responded to these challenges, focusing on the provision of make-up and alternative testing measures.

Methods: We analyzed the availability of make-up and alternative examination options in 82 Japanese medical schools and across 22 national licensing examinations for medical-related professions between 2021 and 2023. Data were collected from official websites of the Ministry of Health, Labour and Welfare, the Ministry of Education, Culture, Sports, Science and Technology, and individual university admissions materials.

Results: Most universities offered make-up examinations or alternative assessment options to students impacted by COVID-19, with a slight decrease in compliance over three years (82% in 2021, 80% in 2022, 76% in 2023). However, none of the national examinations for medical-related professions implemented make-up examinations or similar measures.

Conclusion: Japanese medical schools demonstrated flexibility in adapting examination policies during the pandemic, in contrast to the rigid approach of national licensing examinations. This disparity raises important concerns regarding educational equity and public health preparedness and may also lead to significant financial hardship and career delays for affected examinees. These findings underscore the need for unified, crisis-responsive policies to support students during future health emergencies.

## Introduction

The impact of the novel coronavirus disease 2019 (COVID-19) pandemic has profoundly influenced the learning environment within universities, including medical schools, and the resultant loss of educational opportunities has become a subject of global discourse [1]. Indeed, a 2021 survey among Japanese universities reported that approximately 5% of students who dropped out of university cited COVID-19 as a reason [2]. During the pandemic, educational institutions worldwide were compelled to transition to online platforms [3], and particularly, medical students voiced concerns about the quality of medical education amid the pandemic, especially due to insufficient clinical training [4,5].

In response to this, various initiatives were implemented across medical schools worldwide. In countries such as the United States, United Kingdom, and Denmark, the potential of medical students was highlighted as they were mobilized to support overwhelmed healthcare systems during the COVID-19 pandemic [6-8]. In Italy, the cancellation of the national medical licensing examination allowed for the expedited deployment of medical students to the front, thereby ensuring their educational progression [9]. The University of Debrecen in Hungary addressed students' learning management needs by concurrently offering three self-development courses online, focusing on enhancing study skills, stress management, and COVID-19-related mental health [10].

In Japan, however, the primary national strategy for maintaining educational continuity during the pandemic focused on preserving existing examination schedules and administrative structures, with relatively limited introduction of systemic accommodations or flexible learning reforms. Nevertheless, of note, not all universities were equipped with such supporting systems in Japan during the pandemic. There were reported cases of students being held back due to the lack of make-up classes for COVID-19-related absences and instances where medical students faced mass academic failure due to assessments that deviated from the original syllabus [11]. Additionally, the constitutionally guaranteed freedom of movement was restricted, including limitations on inter-prefectural travel that prevented students from returning home or participating in job-hunting activities, further exacerbating the loss of opportunities for medical students [12].

One of the most significantly impacted areas in the education field has been the opportunity to take examinations, particularly those of entrance exams and national medical licensing exams. In Japan, entrance exams for medical schools are fiercely competitive, with approximately 14 times the number of applicants compared to the available seats each year [13]. Although these exams are generally held once a year, in 2020, there were instances where candidates suspected of COVID-19 infection, without the opportunity to undergo PCR testing, were compelled by public health authorities to voluntarily forgo taking the national examination, which heightened anxiety among examinees [14].

On the other hand, although strict infection-control measures during the pandemic nearly eliminated influenza, Japan experienced its first influenza outbreak in three years in December 2022; moreover, following the relaxation of preventive measures in 2023, other infections also began to increase, indicating that examination disruptions due to infectious diseases will remain a persistent challenge [15-17]. Moving forward, ensuring that examination opportunities are guaranteed for individuals affected by such infectious diseases, including COVID-19, will be increasingly vital.

Nevertheless, in compliance with government directives, examinees are urged to abstain from taking the examination if they are suspected of COVID-19 infection, yet there remains a lack of information regarding accommodations for such examinees. Thus, this study aims to thoroughly investigate the status of make-up exams across various examinations.

## Materials and methods

Medical school entrance examinations in Japan

Medical school entrance examinations in Japan are broadly divided into two categories: national/public universities and private universities, with a total of 82 institutions. Among these, 50 are national or public universities, of which 42 are national and eight are public. On the other hand, there are 31 private universities. In addition to these, there is the National Defense Medical College, which is under the jurisdiction of the Ministry of Defense and functions as a quasi-university.

The National Center Test for University Admissions (renamed as the Common Test for University Admissions starting with applicants in 2021) is mandatory for applicants to national and public universities and may also be required for private university applicants, depending on the method of admission. It is uniformly stipulated that candidates who are unwell may take a make-up exam two weeks later. The National Center Test for University Admissions is held annually in January, followed by individual university exams from January to February. National and public universities primarily offer early admission, later admission, and recommendation-based admission, with applicants being limited to applying to only one university per type of admission. Conversely, private universities do not impose a limit on the number of applications and offer various methods of admission similar to national and public universities, including early and later admissions as well as recommendation-based admissions. However, the specific procedures for these admissions vary by university.

Data collection and analysis

In this study, we investigated the implementation of make-up examinations for medical students in response to COVID-19 at various universities from 2021 to 2023, as well as the response to influenza in 2023. The focus was on the most standard and widely applied examination format for medical school admissions in Japan, specifically those that attract the highest number of applicants. (For Yamanashi University, where the main examination is the General Second Examination, the response to this examination was investigated.) Although COVID-19 was the primary focus of this study, influenza was included as supplementary contextual information to illustrate how universities address infectious-disease-related absences more broadly.

Data for the 2021 academic year were sourced from a website provided by the Ministry of Health, Labour and Welfare (MHLW) for examinees. Data for the 2022 academic year were referenced from the website provided by the Ministry of Education, Culture, Sports, Science and Technology (MEXT) for examinees. Data for the 2023 academic year were gathered from information provided to examinees and student recruitment guidelines published on the websites of each university. For each university, the presence or absence of explicit statements regarding make-up examinations or alternative measures was used to classify the data. Categories such as “information not available” or “alternative measures implemented, but specific details unclear” were assigned only when no clarifying description was provided on the official webpages. Because no single institution publishes these data consistently each year, the information was obtained from the official sources available for each respective year. These data were compiled during the following periods: July 25-August 5, 2022; December 29, 2022-January 16, 2023; and February 14-February 23, 2024. The collected data were categorized into five criteria according to university and examination year. All extracted information was independently double-checked by the two authors (T.M. and Y.K.) on separate days to minimize manual transcription errors.

For COVID-19, the criteria were specifically defined as follows: "make-up examination available (including the alternative selection based on the Common Test for University Admissions, the Center Test for University Admissions, and application documents)", "no make-up examination, but alternative examination available", "alternative measures implemented, but specific details unclear", "no make-up or alternative examination available, with only a refund of the entrance examination fee", and "information on alternative measures not available". The percentage of universities corresponding to each criterion was then calculated. "Make-up examination available" refers to instances where candidates who could not take the scheduled exam were provided with another opportunity to sit for the exam, or where an alternative selection method was employed for those who missed the exam on the day. For example, if a candidate misses the early examination, a make-up examination might be scheduled later as an alternative. "No make-up examination, but alternative examination available" indicates that while no make-up examination is provided, candidates may switch to another available examination method at the same university, even if they had not initially applied for that method. For instance, one common alternative measure was allowing a candidate who missed the early examination due to illness to switch to the later examination. In such cases, the candidate could take the later exam as a substitute opportunity, even if they had not originally applied for that admission round. We also examined the number of universities that mentioned influenza in the information provided to examinees and student recruitment guidelines published on their websites, and within this group, we further investigated the number of universities that implemented make-up examinations or equivalent alternative measures. The percentage of universities corresponding to each criterion was then calculated. Additionally, we investigated the number of examination methods available across all 31 private medical universities (excluding examination methods that could not be substituted, such as recommendation-based admissions, admissions for returnee students, admissions for international students, and admissions for transfer students) and the relationship between these methods and the alternative measures implemented by each university in 2021.

Furthermore, we investigated the implementation of supplementary exams and other measures for COVID-19-infected examinees of all national examinations for medical-related professions in 2021. The 22 national examinations for medical-related professions investigated in this study are as follows: Physician, Dentist, Public Health Nurse, Midwife, Registered Nurse, Radiological Technologist, Clinical Laboratory Technologist, Physical Therapist, Occupational Therapist, Orthoptist, Clinical Engineering Technologist, Prosthetist-Orthotist, Dental Hygienist, Dental Technician, Emergency Medical Technician, Anma-Massage-Shiatsu Practitioner, Acupuncturist, Moxibustion Therapist, Judo Therapist, Speech-Language-Hearing Therapist, Pharmacist, and Registered Dietitian.

## Results

In the entrance examinations for medical schools at various universities, the institutions that provided a "make-up examination available (including the alternative selection based on the Common Test for University Admissions, the Center Test for University Admissions, and application documents)” for candidates infected with COVID-19 were as follows (Table 1): 67 universities (82%) in 2021, 66 universities (80%) in 2022, and 62 universities (76%) in 2023. Universities that offered "no make-up examination, but alternative examination available" were 13 (16%) in 2021, 15 (18%) in 2022, and 16 (20%) in 2023. Furthermore, universities that implemented "alternative measures implemented, but specific details unclear" were 0 (0%) in 2021, 0 (0%) in 2022, and one (1%) in 2023. Those that provided " no make-up or alternative examination available, with only a refund of the entrance examination fee " accounted for one university (1%) in 2021, 0 (0%) in 2022, and 0 (0%) in 2023. Additionally, universities where " information on alternative measures not available " numbered one (1%) in 2021, one (1%) in 2022, and three (4%) in 2023. Regarding influenza, 18 universities (22%) mentioned it in their guidelines, and among these, six universities (7%) implemented make-up examinations or equivalent alternative measures. Moreover, an investigation into the relationship between the number of admission test formats and alternative measures at the 31 private medical universities revealed that all eight universities with only one admission test format conducted make-up examinations, whereas among the 23 universities with two or more test formats, 10 conducted make-up examinations, and 12 offered only alternative examination options without make-up tests. In contrast, none of the national examinations for medical-related professions investigated had implemented any make-up examination measures in 2021.

**Table 1:** Measures Implemented by Medical Schools in Japan for COVID-19-Infected Applicants During Entrance Examinations, Annually from 2021 to 2023

	2021	2022	2023
Make-up examination available (including the alternative selection based on the Common Test for University Admissions, the Center Test for University Admissions, and application documents)	67/82 (82%）	66/82 (80%）	62/82 (76%）
No make-up examination, but alternative examination available	13/82 (16%）	15/82 (18%）	16/82 (20%）
Alternative measures implemented, but specific details unclear	0/82 (0%）	0/82 (0%）	1/82 (1％）
No make-up or alternative examination available, with only a refund of the entrance examination fee	1/82 (1%）	0/82 (0%）	0/82 (0%）
Information on alternative measures not available	1/82 (1%）	1/82 (1%）	3/82 (4%）

## Discussion

This study revealed that 80 out of 82 medical schools in Japan implemented make-up examinations for entrance exams in 2021 in response to the COVID-19 pandemic. Furthermore, during the study period, no universities were found to have changed their policies from offering alternative measures to not offering them. These findings suggest that, during the COVID-19 pandemic, the majority of medical schools in Japan took measures to ensure that candidates who were infected with or suspected of being infected with the virus were afforded an opportunity to take their exams. Additionally, it was observed that among private medical schools with two or more types of admission test formats, a greater number opted for alternative examination measures, such as substitute exams, rather than make-up exams, compared to universities with only one admission test format. Although it would be ideal for universities to provide make-up exams, which are comparable to the originally scheduled exams, for candidates who were unable to take their exams due to the impact of COVID-19, it is commendable that nearly all universities, except one, offered substitute exams to ensure a fair opportunity to take the test.

However, there are some concerns regarding the handling of candidates suspected of being COVID-19 positive on the day of the exam. According to the "Guidelines for the Implementation of University Entrance Examinations in Response to COVID-19 for the Academic Year 2022" issued by the MEXT, "Examinees with symptoms such as fever or cough should refrain from taking the exam if their body temperature is 37.5 degrees Celsius or higher on the day of the exam and should consider taking a make-up exam. Examinees with symptoms but no fever above 37.5 degrees Celsius should inform the exam proctor of their condition [18].” This indicates that if a candidate exhibits symptoms such as fever or cough on the day of the exam, they would be prohibited from taking the exam in a separate room if their temperature exceeds 37.5°C, even without virological confirmation. This may be related to Japan's long-standing practice during the pandemic of categorizing individuals who had contact with COVID-19-positive individuals as "close contacts" without conducting PCR or antigen tests and restricting their activities [19].

In this study, we investigated the responses of medical schools to examinees who were unable to take their entrance exams due to the impact of COVID-19. The findings indicate that most medical schools have implemented alternative measures such as supplementary or rescheduled exams. In contrast, none of the 22 national medical examinations offered supplementary tests. Given that the MHLW stipulates that examinees who are infected with COVID-19 and are hospitalized, undergoing home treatment, or in accommodation for recovery are not permitted to take the exams due to the risk of infection to others, it seems necessary to provide alternative examination opportunities [20]. Particularly as the Omicron variant of the coronavirus has increasingly affected younger populations, and with studies from the United States indicating that over 40% of COVID-19 cases involved false reporting, it is easy to imagine that the current measures may lead students to conceal their infections and attend in person, creating a moral hazard driven by the absence of make-up examinations and potentially contributing to cluster outbreaks [21,22].

In contrast to the flexible accommodations implemented by individual medical schools, concerning healthcare profession examinations, none of the 22 national examinations for medical-related professions adopted supplementary measures. This disparity likely reflects structural differences in how examinations are administered: universities possess greater autonomy and maintain direct relationships with applicants, allowing for flexible accommodations, whereas national licensing examinations are centrally administered and prioritize standardization over individual arrangements. The MHLW justified this by citing the logistical challenges of rapidly creating supplementary test questions [23]. This justification is questionable, particularly given the precedent set by the national nursing examination in 2014, which was rescheduled due to severe weather conditions [24]. Moreover, conversely, Japan’s rigid approach to national licensing examinations was particularly striking compared with the more flexible measures adopted in other countries. In the United Kingdom, among medical schools, 43% that had conducted the OSCE before lockdown did so, while the remaining schools either canceled the exams or the OSCE stations requiring interaction with simulated or real patients [25]. Furthermore, while 55.9% of final-year medical students' written exams were held as usual, other universities either canceled the exams, reduced the number of exams or duration, or adapted by conducting online exams [25]. In the United States, the USMLE Step 1 examination was canceled from 2020 to 2021, and the rescheduling process was haphazard, adversely affecting many medical students scheduled to take the exam [26]. The inability to take national examinations due to COVID-19 implies a delay of at least one year in starting work, which results in significant disadvantages in terms of both finances and career. For instance, the income will be delayed by a year, with unexpected expenses likely arising during the income-free year. In terms of career, a one-year delay means a corresponding reduction in clinical experience as a physician compared to others, presenting considerable disadvantages.

One of the limitations of this study is that it does not provide information on the number of students who were eligible for supplementary exams. Future research could address this limitation by directly surveying universities to determine how many students actually utilized these make-up or alternative examinations and to evaluate the effectiveness of these measures. Additionally, as the survey was conducted manually, there may be errors despite our utmost care. Furthermore, because this study is descriptive in nature, the presence of a policy does not indicate its actual implementation or effectiveness, nor can the study determine whether the accommodations provided were adequate for examinees. These aspects could be evaluated in future research through institutional surveys or administrative data, which would allow a more detailed assessment of policy uptake and impact. Nevertheless, the results offer crucial insights into ensuring equal opportunities for students facing infection-related barriers to examination access amidst the COVID-19 pandemic in Japan, aligning with broader practices to guarantee uniform access to examination opportunities. Moreover, this assessment provides important implications for responding to potential future pandemics, including new variants of COVID-19 or other emerging infectious diseases.

## Conclusions

This study revealed that while many medical schools in Japan have implemented various accommodations for applicants infected during the COVID-19 pandemic, the response in national medical examinations has consistently been inadequate. When exams are deferred to the following year, delays in graduation or licensure can result in substantial practical burdens, including prolonged living expenses and a delayed start of income, which may disadvantage students and increase the risk of mental health difficulties. Furthermore, absences due to infection constitute force majeure under the Infectious Disease Law, making this not just a personal issue but a significant public health challenge. Therefore, it is imperative that relevant agencies collaboratively establish clear, crisis-responsive examination policies, such as standardized make-up examinations, flexible alternative testing options, and transparent communication protocols, to prevent disadvantaged outcomes for infected examinees and to enhance preparedness for future pandemics.
